# A Sensitive Assay for Virus Discovery in Respiratory Clinical Samples

**DOI:** 10.1371/journal.pone.0016118

**Published:** 2011-01-24

**Authors:** Michel de Vries, Martin Deijs, Marta Canuti, Barbera D. C. van Schaik, Nuno R. Faria, Martijn D. B. van de Garde, Loes C. M. Jachimowski, Maarten F. Jebbink, Marja Jakobs, Angela C. M. Luyf, Frank E. J. Coenjaerts, Eric C. J. Claas, Richard Molenkamp, Sylvie M. Koekkoek, Christine Lammens, Frank Leus, Herman Goossens, Margareta Ieven, Frank Baas, Lia van der Hoek

**Affiliations:** 1 Laboratory of Experimental Virology, Department of Medical Microbiology, Center for Infection and Immunity Amsterdam (CINIMA), Academic Medical Center of the University of Amsterdam, Amsterdam, The Netherlands; 2 Department of Clinical Epidemiology, Biostatistics and Bioinformatics, Bioinformatics Laboratory, Academic Medical Center of the University of Amsterdam, Amsterdam, The Netherlands; 3 Department of Genome Analysis, Academic Medical Center of the University of Amsterdam, Amsterdam, The Netherlands; 4 Department of Medical Microbiology, University Medical Center Utrecht, Utrecht, The Netherlands; 5 Department of Medical Microbiology, Leiden University Medical Center, Leiden, The Netherlands; 6 Laboratory of Clinical Virology, Department of Medical Microbiology, Center for Infection and Immunity Amsterdam (CINIMA), Academic Medical Center of the University of Amsterdam, Amsterdam, The Netherlands; 7 Department of Medical Microbiology, Vaccine and Infectious Disease Institute, Universiteit Antwerpen, University Hospital Antwerp, Antwerp, Belgium; 8 Department of Data Management, Julius Center for Health Sciences and Primary Care, University Medical Center Utrecht, Utrecht, The Netherlands; University of Hong Kong, Hong Kong

## Abstract

In 5–40% of respiratory infections in children, the diagnostics remain negative, suggesting that the patients might be infected with a yet unknown pathogen. Virus discovery cDNA-AFLP (VIDISCA) is a virus discovery method based on recognition of restriction enzyme cleavage sites, ligation of adaptors and subsequent amplification by PCR. However, direct discovery of unknown pathogens in nasopharyngeal swabs is difficult due to the high concentration of ribosomal RNA (rRNA) that acts as competitor. In the current study we optimized VIDISCA by adjusting the reverse transcription enzymes and decreasing rRNA amplification in the reverse transcription, using hexamer oligonucleotides that do not anneal to rRNA. Residual cDNA synthesis on rRNA templates was further reduced with oligonucleotides that anneal to rRNA but can not be extended due to 3′-dideoxy-C6-modification. With these modifications >90% reduction of rRNA amplification was established. Further improvement of the VIDISCA sensitivity was obtained by high throughput sequencing (VIDISCA-454). Eighteen nasopharyngeal swabs were analysed, all containing known respiratory viruses. We could identify the proper virus in the majority of samples tested (11/18). The median load in the VIDISCA-454 positive samples was 7.2 E5 viral genome copies/ml (ranging from 1.4 E3–7.7 E6). Our results show that optimization of VIDISCA and subsequent high-throughput-sequencing enhances sensitivity drastically and provides the opportunity to perform virus discovery directly in patient material.

## Introduction

Respiratory tract infection is the most common cause of hospitalization of children below the age of 5 years [1], [2]. In 5–40% of these hospitalizations no infectious agent can be identified but it is suspected that a viral infection is involved [3]–[5]. In these cases a yet unknown virus might be the cause of respiratory illness.

In the last decades several viral discovery methods have been developed which can detect viruses without knowledge of the genome sequence. We have previously used virus discovery cDNA-AFLP (VIDISCA) to discover the human coronavirus NL63 (HCoV-NL63) [6] and we were the first to describe human parechovirus type 5 and 6 in the Netherlands using the same technique [7]. In the VIDISCA assay viral genomes (which are (reverse-) transcribed into double stranded DNA) are digested with restriction enzymes. The enzymes digest short (4 nucleotides) recognition sequences that are present in virtually all viruses. After ligation of adaptors, the digested fragments are PCR amplified with adaptor-specific primers. The assay is user-friendly however the sensitivity of the assay is low. At least 1 E6 genome copies/ml of a virus in a background that is low in competitor RNA/DNA are needed. These conditions are generally only met when virus culture supernatant is used. In clinical respiratory samples like nasopharyngeal swabs in universal transport medium (UTM) various amounts of competitor RNA/DNA from disrupted cells/bacteria can be present. Ribosomal RNA, which is ∼80% of the total cellular RNA, is one of the biggest problems due to its high copy number and its stability within ribosomes. In particular RNA viruses are difficult to discover since in these cases a reverse transcription is needed, which will enable rRNA to act as competiting nucleic acid sequences.

One research group has addressed the problem of competing rRNA [8]. Endoh et al showed that reverse transcription with 96 hexamers that can not anneal to rRNA, decreases the amount of background amplification and enhances the sensitivity of a virus discovery assay. We evaluated the benefit of the non-rRNA-hexamers in VIDISCA. Furthermore, we evaluated whether the choice of the restriction enzyme can decrease rRNA amplification. Finally, specific blocking of rRNA reverse transcription by rRNA recognizing oligo's that contain a 3′ dideoxy-C6 modification (which can not be extended), further inhibits cDNA synthesis of the target. All three steps to decrease the effect of inhibitor rRNA are presented in this paper. Furthermore we monitored the performance of the optimized amplification in a high throughput sequencing setting, by combining VIDISCA with Roche 454 GS FLX Titanium sequencing.

## Results

### VIDISCA with decreased amplification of background rRNA

Respiratory samples contain non-viral nucleic acids that interfere in virus discovery techniques like VIDISCA. It is relatively easy to decrease the influence of background bacterial or human DNA and mRNA by centrifugation and DNase/RNase treatment, but ribosomal RNA (rRNA) is difficult to eliminate because the ribosomal proteins protect the rRNA inside the ribosomes. Instead of degrading the competing rRNA, it is an option to adjust the amplification procedure during VIDISCA, such that rRNA amplification decreases. The method can be adjusted at several levels: 1) non-rRNA-annealing-primers can be used during reverse transcription 2) a choice for certain restriction enzymes can be made to diminish the chance of rRNA digestion and subsequent amplification, and 3) rRNA-blocking oligos can be used during the reverse transcription to halt cDNA synthesis on an rRNA template.

#### 1) non-rRNA-hexamers in the reverse transcription reaction

**Figure 1:**
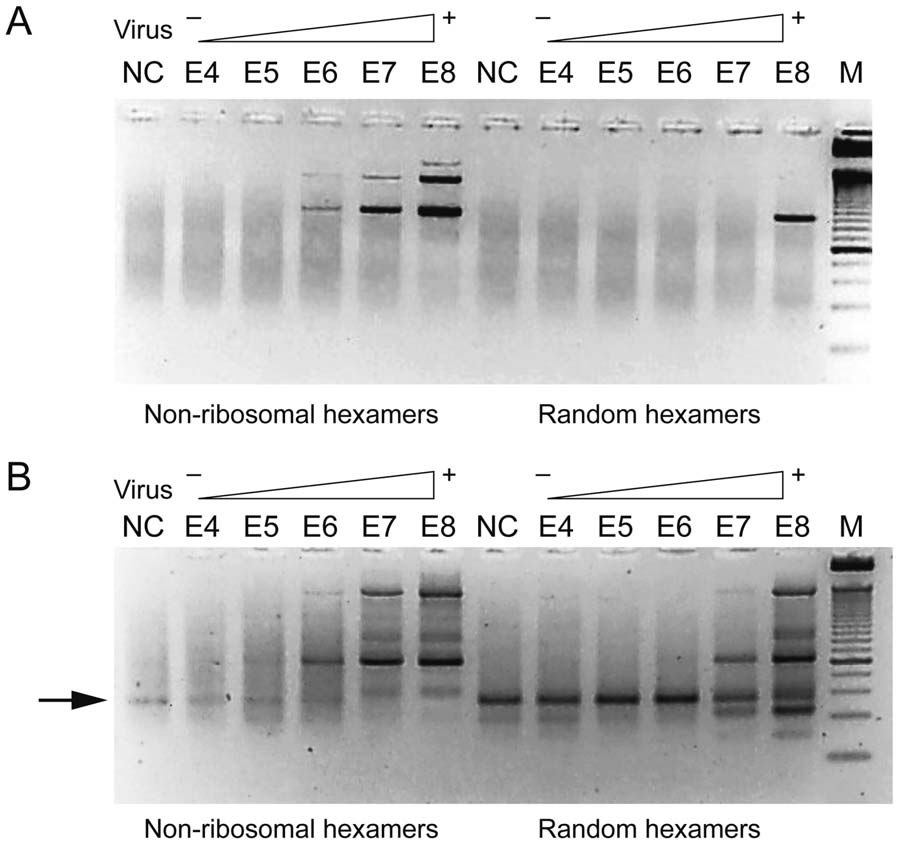
Enhanced viral RNA amplification in VIDISCA using non-ribosomal hexamers during reverse transcription. VIDISCA fragments are visualized on a 3% metaphor gel. A dilution series of echovirus 18 was used and the concentration per ml is indicated above each lane. NC = negative PBS control, M = 25 bp marker.(a) VIDISCA products were generated with primers Hinp-A/Mse-C. The viral fragments are 167 bp, 296 bp and 382 bp in size. (b) VIDISCA products amplified with primers Hinp-A/Mse-A. The product originating from rRNA (70 bp) is indicated by an arrow.

Endoh and colleagues designed a mix of 96 hexamers that do not or hardly target rRNA but can amplify all known viruses by RT-PCR [8]. These non-rRNA-hexamers were tested in VIDISCA by using a dilution range of human echovirus 18 culture supernatant (1 E8–1 E4 copies/ml), a virus harvest of which we established that it contains competitor rRNA. The cDNA was produced either with normal hexamers (containing the 4096 variants) or non-rRNA-hexamers. Viral sequences could be detected in samples with a concentration of 1 E6 to 1 E8 viral genomic RNA copies/ml (see in figure 1A) in case non-rRNA-hexamers are used in the RT reaction, whereas the sample that was treated with the normal random hexamers was only positive in the highest concentration (1 E8 copies/ml). Moreover, 3 viral fragments were amplified in the non-rRNA-hexamer amplification, whereas only 1 viral fragment was amplified with the standard procedure (figure 1A). Figure 1B shows that the enhanced sensitivity is caused by reduced competitor rRNA amplification, since the PCR fragment that originates from rRNA is notably reduced (arrow in figure 1B).

To quantify the inhibition of rRNA amplification we performed various real-time PCRs targeting cDNA of 28S rRNA and 18S rRNA using 2 nasopharyngeal swabs (I and II) as input. Both samples contained high concentrations of rRNA. The samples were reverse transcribed with either the complete set of hexamers or the non-rRNA-hexamers. With non-rRNA-hexamers substantially lower amounts of rRNA-derived cDNA was generated with on average more than 1 log decrease, compared to the samples treated with random hexamers (see table 1). We observed that the decreased cDNA synthesis on the 3700-region of 28S rRNA and 1000-region of 18S rRNA was considerable (∼3,5 Ct = 1 log), however, not as strong as the decrease at regions 40–110 and 1780–1880 of 28S rRNA (almost 2 log decrease, table 1). Inspecting the non-rRNA-hexamers revealed that this phenomenon can be explained by residual priming by the non-rRNA-hexamers. Although the primers are designed to anneal not or hardly to rRNA, some do perfectly match with human rRNA, especially in the region 3800 to 4000 (position 3803, 3840, 4040), and the same for 18S rRNA region 1100 till 1200 (position 1121, 1123, 1134, 1185, 1187, 1207). However, in the regions where we show strong decrease in rRNA cDNA synthesis (40–110 128S rRNA and 1780–1880 18S rRNA), non-rRNA-hexamer can not anneal at the 3′site at close vicinity (position 1613 and 2272 respectively). One might suggest expelling the 8 hexamers that anneal at the abovementioned locations to further enhance the benefit of non-rRNA cDNA synthesis. However, Endoh et al designed the non-ribosomal hexamers such that amplification of viruses is not hampered, therefore we recommend using all 96 Endoh-designed non-rRNA hexamers.

**Table 1:** Decrease of cDNA synthesis on rRNA templates.

	Decrease rRNA-cDNA synthesis with non-rRNA-hexamers^a^		Decrease rRNA-cDNA synthesis with rRNA-blocking oligo's^b^		Total decrease^c^	
Sample number:	I		I	II	I	II
**Region in rRNA**						
40–110 28S	97%	96%	66^d^%	36^d^%	**98**%	**98**%
1780–1880 28S	98%	96%	7^e^%	0^e^%	**95**%	**96**%
3700–3800 28S	81%	83%	30^f^%	39^f^%	**87**%	**90**%
930–1050 18S	75%	84%	51^g^%	0^g^%	**88**%	**83**%

a In comparison to cDNA synthesis with all 4096 random hexamers.

b In comparison to cDNA synthesis without rRNA-blocking oligo's.

c In comparison to cDNA synthesis with all 4096 random hexamers and without rRNA-blocking oligo's.

d binding region for blocking oligo 4-Morrna.

e no rRNA-blocking oligo directed to this 1780–1880-region of 28S rRNA was added.

f binding region for blocking oligo 3-Morrna.

g binding region for blocking oligo 1-Morrna.

To check whether viral amplification is not hampered by using the non-rRNA-hexamers for cDNA synthesis we performed real-time PCRs on cDNA of HCoV-NL63, echovirus 18, and human coxsackievirus A16 virus culture supernatant. In all cases the cDNA synthesis with non-rRNA-hexamers occurs as efficient as normal hexamers, as no difference in virus specific real time PCRs was noted (Table 2). The same has been demonstrated by Endoh*et al* for SARS-CoV and bovine PIV-3 control viruses [8].

**Table 2:** No decrease in viral genome amplification with random hexamers versus non-ribosomal hexamers.

Virus	Reverse transcription- primers	Ct Values
HCoV-NL63	Random hexamers	22.31
	Non-ribosomal hexamers	23.12
Echovirus 18	Random hexamers	14.84
	Non-ribosomal hexamers	14.90
Coxsackievirus A16	Random hexamers	22.16
	Non-ribosomal hexamers	20.56

#### 2) non-rRNA targeting restriction enzymes during digestion

**Figure 2:**
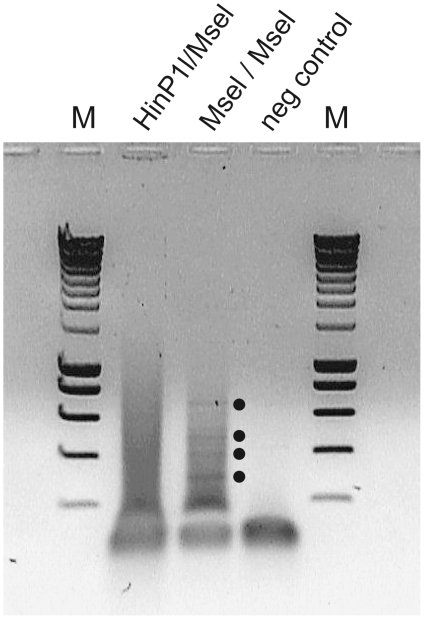
Enhanced amplification of viral fragments using one restriction enzyme in VIDISCA. Visualization of VIDISCA fragments digested with *HinP1-I+MseI* or *MseI* alone. VIDISCA fragments are visualized on a 1% agarose gel, which were generated after a single first round PCR of 40 cycles. The dots indicate viral fragments which were only visible with *MseI* digestion.

The original VIDSICA method described in 2004 is based on amplification after digestion with 2 restriction enzymes (*Hinp1-I* and *MseI*) [6]. Investigation of human rRNAs revealed that 28S rRNA contains a very high number of *Hinp1-I* recognition sites (85, see table 3), but relatively low frequency of *MseI* restriction sites. The high frequency of *HinP1-I* digestion in 28S rRNA and the generation of a massive amount of small digested fragments likely interferes in the VIDISCA-ligation. VIDISCA can also be performed with only one restriction enzyme, the only adaptation needed is the addition of 2 different adaptors that both can ligate to *MseI* digested fragments. We checked our hypothesis by digesting coxsackievirus B4 culture supernatant with only *MseI* in comparison to the *Hinp1-I/MseI* combination, and evaluated the efficiency of viral genome amplification in a single PCR. We observed a strongly reduced background amplification in case only *MseI* was used in VIDISCA (Figure 2, dots all indicate viral fragments).

**Table 3:** Theoretical VIDISCA amplifiable fragments in human rRNA, and number of restriction sites.

rRNA	HinP1I nr of recognition sites	MseI nr of recognition sites	HinP1I×MseI nr of fragments^a^	MseI×MseI nr of fragments^a^
5.8 S rRNA	0	1	0	0
18 S rRNA	11	12	7	9
28 S rRNA	85	8	8	4

a only fragments larger than 50 nt and smaller than 600 nt are counted.

#### 3) rRNA-blocking oligos in the reverse transcription reaction

**Figure 3:**
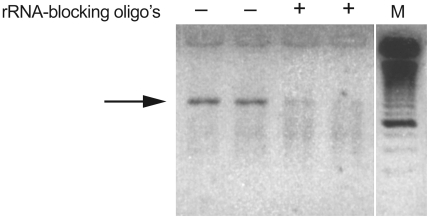
rRNA-blocking oligo's decrease rRNA-cDNA synthesis in VIDISCA. VIDISCA fragment of ribosomal RNA visualized on a 3% metaphor gel. A nasopharyngeal washing was used as input for VIDISCA with or without blocking oligo's. Lane 1 and 2 are without blocking oligo's whereas lane 3 and 4 are with blocking oligo's, M = 25 bp marker. The arrow indicates the rRNA fragment of which the amplification was decreased.

To improve the sensitivity of VIDISCA even further we designed oligonucleotides to block amplification of ribosomal RNA. These oligonucleotides were designed to anneal specifically to 18S and 28S rRNA and contain a 3′ dideoxy C6 amino modification to inhibit the elongation and thus the amplification of rRNA-derived cDNA. These so called rRNA-blocking oligo's were designed on the most prevalent rRNA sequences retrieved from VIDISCA experiments with nasopharyngeal swabs. To test the inhibitory capacity of the blocking oligo's we performed VIDISCA with a nasopharyngeal sample as input. Blocking oligo's were added during reverse transcriptase reaction, and inhibition was observed when blocking oligo's were added (indicated as arrow in figure 3). Sequencing of the inhibited PCR products confirmed that they were derived from rRNA indicating that the blocking oligo's can reduce the amplification of rRNA.

In addition we performed a real-time RT-PCR targeting 18S and 28S rRNA. As input 2 nasopharyngeal samples were used (same samples that were used with the non-rRNA annealing hexamers). We monitored cDNA synthesis via real time PCRs at 3 regions of 28S rRNA and 1 region of 18S rRNA. The choice for these regions to monitor the rRNA-cDNA reverse trancription efficiency was based on the VIDISCA fragments of which we know that they are generated in VIDISCA amplification. Three of the 4 regions are targeted by the rRNA-blocking oligo's. On average a 50% reduction of rRNA amplification was noticed at the regions that were targeted by the rRNA-blocking oligo's (see Table 1). Of note, the reduction was not visible in the fragment that was not targeted by a blocker (1780–1880 of 28S rRNA), which is as expected. One exception was observed however. In sample II no diminished signal was observed at 18S rRNA 1000-region. This sample had extremely high concentrations of rRNA (Ct value 16), thus we investigated whether the rRNA-blocking oligo's would work better in this sample when higher concentrations of the blockers were used. Indeed, with 25 µM and 50 µM a decrease in signal was noted (36.9% decrease and 70.1% decrease, respectively) indicating that in some samples a concentration of 10 µM might be suboptimal. However, to diminish the chance of unspecific blocking of viral RNA, we prefer the 10 µM concentration of rRNA-blocking oligo's. With this concentration we observed no decrease in cDNA synthesis on HCoV-NL63 and coxsackievirus B4 (measured by real-time RT-PCR, (table 4) ).

**Table 4:** No inhibition of viral genome amplification with rRNA-blocking oligo's.

Virus	rRNA-blocking oligo's	Ct Values
HCoV-NL63	+	14.8
	−	13.6
Coxsackievirus B4	+	18.5
	−	19.0

### VIDISCA combined with high throughput sequencing

In figure 1 it is shown that the sensitivity of VIDISCA reaches 1 E6 viral genome copies/ml. Although this is an improvement, this detection limit might be too low to detect viruses directly in clinical samples. The concentration of respiratory viruses in nasopharyngeal swabs is in the main below 1 E6 copies/ml, and we can assume that a yet unknown virus will be present in similar concentrations. Thus additional improvement of the VIDISCA-sensitivity is needed. High throughput sequencing is a relatively new method allowing millions of nucleotides to be sequenced in only one run (pyrosequencing). One of these devices is the 454 FLX/Titanium system of Roche which can generate over 1.000.000 DNA fragments of approximately 500 nucleotides per run. By generating thousands of clonal amplified sequences from a single sample, a viral minority can be detected. The VIDISCA technique can easily be adapted for 454-FLX sequencing (VIDISCA-454 method). The anchors that are ligated to the digested fragment can be designed to contain the “A” and “B” primer sequence that are needed for clonal amplification in an emulsion PCR to be used as input for 454 FLX sequencing.

However, VIDISCA-454 only becomes cost-effective in case a few thousand sequences are sufficient for virus detection, as one 454 plate can then be used to analyze 56 samples (roughly 200 € per sample). In that view VIDISCA-454 benefits strongly from the aforementioned reduction in rRNA amplification since fewer sequences are needed to detect a viral sequence.

We monitored the efficiency of VIDISCA-454 in 18 nasopharyngeal swabs that contain known viruses. Only one third of a 454 picotiterplate was used, to check whether indeed a few thousand sequences are enough for virus detection. Samples were selected randomly from a large sample set collected during the GRACE study, a large EU financed study on acute cough and antibiotic use in adults. The 18 samples were assigned positive via specific diagnostic PCRs, but supplied to us double blind to ensure unbiased sequence analyses. Each sample was processed with its own identifier sequence that allows pooling during emulsion PCR. VIDISCA-454 products were visualized on agarose gel and fragments were cut from gel at different size regions (*200–300*, *300–500 and 500–700 bp*). Samples were run on 1.3 regions of a 4 regions Picotiterplate for the 454 Titanium system (per region 14 MID tagged samples were pooled) and processed according to the small volume emulsion PCR. In total 202.975 reads were generated of which 4406 were viral (2.2%). In 11 out of 18 samples viral sequences could be identified which all matched with the respiratory virus that was found in diagnostic PCRs (Table 5). The frequency of viral sequences per sample ranged between 0.01% and 40.5% (Table 5). The median viral load in the VIDISCA-454 positive samples was 7.2 E5 viral genome copies/ml (ranging from 1.4 E3–7.6 E6 genome copies/ml). Detection was correlated to input viral load since the very low load samples remained negative in VIDISCA-454 (median viral genome concentration in VIDISCA-negative samples 3.5 E3; range 6.0 E2–1.1 E5). For most VIDISCA-454 positive samples large genome coverage was observed, see table 5.

**Table 5:** Respiratory virus detection with VIDISCA-454.

Sample nr	Respiratory virus	Viral load copies/ml	Result VIDISCA-454	Nr of reads	Nr of viral reads	% viral reads	% genome coverage
A0211	Human PIV-1	1.2 E3	-	32671	0	<0.003%	
D0424	RSV	2.4 E4	-	3437	0	<0.02%	
E1573	Influenza B	6.1 E4	Influenza B	4262	2	0.04%	0.8%
A2829	Influenza A	8.9 E5	Influenza A	9924	14	0.14%	11%
E0061	HCoV-NL63	1.0 E4	HCoV-NL63	8641	3	0.03%	0.8%
I1647	RSV	3.3 E6	RSV	3497	167	4.8%	4%
I4335	Influenza B	1.5 E4	-	2283	0	<0,04%	
O1189	HRV	1.2 E5	-	4030	0	<0.02%	
E0117	Influenza A	6.0 E2	-	2449	0	<0.04%	
I0555	Adenovirus	1.4 E3	Adenovirus	13478	13	0.1%	4%
I2193	RSV	1.2 E6	RSV	16701	577	3.5%	71%
I4363	Influenza B	1.5 E6	Influenza B	15595	459	2.9%	30%
O2967	Influenza B	2.0 E5	Influenza B	8132	14	0.2%	11%
S2719	HCOV-OC43	7.6 E6	HCoV-OC43	7437	3014	40.5%	79%
B0702	HCoV-OC43	3.5 E3	-	10170	0	<0.01%	
F1308	Influenza A	1.4 E3	-	9556	0	<0.01%	
H1940	Influenza A	1.9 E3	Influenza A	8362	1	0.01%	0.3%
I3747	HCoV-OC43	7.2 E5	HCoV-OC43	11691	114	1.0%	22%

## Discussion

Nowadays molecular techniques are becoming the standard for the discovery of new viruses. Some methods use a conserved region for universal primer design, based on the known viral genomes [9]–[11]. These methods are applicable to specific virus families, but cannot be used for all viruses. Furthermore, some yet unknown viruses could be too diverse and therefore remain negative in these kind of detection techniques [7]. Sequence independent amplification methods, such as VIDISCA and random-PCR, can identify viral sequences without prior knowledge of a viral genome. Unfortunately, the detection of unknown viral pathogens in respiratory clinical material is difficult with these sequence independent virus discovery methods because of low viral load and high background nucleic acids in these samples. During the last years sequence independent virus discovery techniques were mostly used with virus culture supernatant, as they contain high concentrations of viral genomes [6], [12], or to discover previously unknown DNA viruses [13]–[15]. So far no study has been able to identify novel human respiratory RNA viruses with sequence independent amplification techniques. Thus sequence independent amplification techniques like VIDISCA have to be optimized to allow discovery without requiring a culture amplification step.

In the current study we increased the sensitivity of VIDISCA by 1) reducing background rRNA amplification, and 2) by increasing the number of sequences obtained from a sample. We managed to unfavor rRNA amplification by adjusting the reverse transcription step. Utilization of primers during cDNA synthesis that poorly recognize rRNA, in combination with the addition of oligo's that halt cDNA synthesis on rRNA templates successfully decreased interfering background amplification. Additionally, using a single restriction enzyme with low numbers of recognition sites in 28S rRNA provided further reduction of useless and interfering amplification. Thus all steps increased the ratio of viral genome versus rRNA amplifications, and the benefit was shown in VIDISCA-high throughput sequencing of clinical samples containing known viruses. In the majority of clinical samples the virus was easily identified by VIDISCA-454 (11 of 18). In two cases even an input of 140 and 190 genome copies of an adenovirus and influenza A virus could be detected by VIDISCA-454. Ideally, old-protocol VIDISCA-454 (two restriction enzymes, random hexamers and no rRNA-blocking oligo's) should have been compared with optimized VIDISCA-454. However, this comparison is regrettably not possible due to limitation of the respiratory clinical specimens that we used. Thus we rely on all the reconstructions and monitoring performed with normal VIDISCA.

As mentioned above, the use of one restriction enzyme (*MseI*) diminished background rRNA amplification. There is one additional advantage of single restriction enzyme usage. In the traditional VIDISCA two restriction enzymes were combined (*MseI* and *HinP1-I*) and only fragments that have one restriction site on the 5′ site and the other in the 3′ site are amplified after ligation. Such VIDISCA amplification is restricted in case one of the two enzymes has few recognition sites, or when the position of the sites is not optimal (too far or too close from each other). By using only one restriction enzyme, large parts of the genome would be divided in amplifiable products, provided that the fragment size is between 50 and 600 bp. In case of single restriction enzyme digestion, both anchors can potentially ligate to both *MseI* generated sticky end but only AB or BA containing fragments can be used for sequencing. This might give the suggestion that 50% of the VIDISCA products are ineffective as they contain the same adaptor (AA and BB). However, the fragments containing 2 different primers are preferentially amplified in the PCR, since an AA or BB fragment has a disadvantage that 5′ and 3′ ends anneal to each other which interferes with primer annealing. We definitely observed the higher chance of amplification of several genome segments when only one restriction site is used. Remarkably high genome coverage was noted in several samples (reaching >70% for the samples containing RSV and HCoV-OC43), a coverage which could never be achieved in case two restriction enzymes were used in amplifications.

Other groups have used high throughput sequencing for virus discovery as well. In one paper the viral community in an Antarctic lake was described [16]. Lopez-Bueno *et al.* collected water in spring and late summer from a fresh water lake (Limnopolar lake) in Antarctica and used high throughput sequencing to study the viral community in a location hardly visited by larger eukaryotes. For the first time a large amount of sequence data was retrieved from this isolated place which led to the identification of at least 12 viral families of which two are claimed to represent new families. Their results show the enormous possibilities for virus discovery and high throughput sequencing. The authors also address a large amount of unknown sequences present in their data set. We also observed the presence of unknown sequences within our data set. It could be that these sequences are derived from yet unknown viruses, or it could be that the sequences are part of a genomic sequence from a known organism, e.g. a bacterium of which not the complete genomic sequence is present in the Genbank databases. Thus care should be taken to assign sequences as potentially viral, since so many organisms have not been fully sequenced.

There are several advantages of high throughput sequencing in comparison to BigDye terminator sequencing. First of all, with high throughput sequencing and pooling of samples that carry their own recognition sequence the VIDISCA cost per sample is reduced, since selective VIDISCA-PCR, metaphor agarose gel visualization, purification of fragments from gel, TA cloning, colony PCR and subsequent BigDye sequencing can all be omitted. Secondly, the amount of sequence data received from a single sample is higher than what can be achieved in standard VIDSCA, thus increasing the chances of identifying an unknown virus. This method opens new opportunities for virus discovery, not only in respiratory samples of undiagnosed respiratory infection, but also in diseases such as Amyotrophic lateral sclerosis (ALS), Kawasaki disease (KD) and Multiple sclerosis (MS). For these syndromes a viral pathogen has been suggested [17]–[19] but could not be confirmed so far. With VIDISCA-454 it is now possible to investigate samples from these patients for unknown viruses.

## Materials and Methods

### Ethics Statement

Patients were randomly chosen from the large European EU-financed GRACE study (https://www.grace-lrti.org). Ethics review committees in each country approved the study, Cardiff and Southampton (United Kingdom): Southampton & South West Hampshire Research Ethics Committee A; Utrecht (Netherlands) Medisch Ethische Toetsingscommissie Universitair Medisch Centrum Utrecht; Barcelona (Spain) Comitè ètic d'investigació clínica Hospital Clínic de Barcelona; Mataro (Spain): Comitè d'Ètica d'Investigació Clínica (CEIC) del Consorci Sanitari del Maresme; Rotenburg (Germany) Ethik-Kommission der Medizinischen Fakultät der Georg-August-Universität Göttingen, Antwerpen (Belgium): UZ Antwerpen Comité voor Medische Ethiek; Lodz, Szeczecin, and Bialystok (Poland): Komisja Bioetyki Uniwersytetu Medycznego W Lodzi; Milano (Italy) IRCCS Fondazione Cà Granda Policlinico; Jonkoping (Sweden): Regionala etikprövningsnämnden i Linköping; Bratislava (Slovakia): Etika Komisia Bratislavskeho; Gent (Belgium): Ethisch Comité Universitair Ziekenhuis Gent; Nice (France) Comité de Protection des Personnes Sud-Méditerranée II, Hôpital Salvator; Jesenice (Slovenia): Komisija Republike Slovenije za Medicinsko Etiko. Written informed consent was provided by all study participants.

### Clinical samples and viruses

HCoV-NL63, echovirus 18, human coxsackievirus A16 and human coxsackievirus B4 were cultured on an epithelial monkey kidney cell line (LLC-MK2 [6]) in MEM Hank's/Earle's (2∶1) medium (Invitrogen) with 3% inactivated fetal bovine serum (FBS; Cambrex Bio Science). Both media were supplemented with penicillin (0.1 mg/ml) and streptomycin (0.1 mg/ml) (Duchefa Biochemie). Viruses were harvested on day 2 except human coronavirus NL63 (HCoV-NL63) which was harvested at day 7.

During the GRACE study, a large EU financed study on acute cough and antibiotic use in adults consulting their general practitioner, flocked nasopharyngeal swabs (Copan) in universal transport medium (UTM) were collected from all patients. Eighteen of these nasopharyngeal specimens were randomly selected (double blind) and included in this study and proven positive by specific diagnostic PCR's for either human rhinovirus (HRV), respiratory syncytial virus (RSV), human coronavirus OC43 (HCoV-OC43), HCoV-NL63, Influenzavirus A, Influenzavirus B, parainfluenzavirus 3 (PIV3) or adenovirus. The diagnostics for the respiratory viruses were determined by in-house multiplex real-time PCR assays [20]–[22], all primers and probes are available on request. Viral loads were determined by virus-specific quantative real time PCRs using standard curves based on plasmids containing the virus sequence of interest (details available on request).

### Real time RT-PCR for enterovirus, HCoV-NL63 and rRNA

Nucleid acids were extracted by Boom isolation [23]. Elution of nucleic acids was performed in sterile H_2_O or in 10 µM of rRNA-blocking oligonucleotides (2 µM each, see below). The reverse transcription was performed as described [6] with the adjustment that in some cases 25 ng of random hexamers (Amersham Biosciences) or non-ribosomal hexamers were used. Enterovirus real-time PCR was performed to quantify the efficiency of echovirus 18, human coxsackievirus A16 and human coxsackievirus B4 reverse transcription reactions, whereas a specific HCoV-NL63 real time PCR was performed to quantify the HCoV-NL63 reverse transcription efficiency [24], [25]. Ribosomal RNA real time PCR was performed with the primers below, and the Quantifast SYBR Green PCR kit (Qiagen). Real-time PCR with primerset 5/6 was additionally run with a probe (rRNA28S_3674 5′-FAM-GGGTGTTGACGCGATGTGATTTCT-TAMRA-3′) and the platinum quantitative PCR Supermix-UDG system (Invitrogen).

1. rRNA28S_40F 5′-TCAGATCAGACGTGGCGACCCGCTG-3′
2. rRNA28S_110R 5′-CGCTGGGCTCTTCCCTGTTCACTC-3′
3. rRNA28S_1780F 5′-TGGGTAAGAAGCCCGGCTCGCT-3′
4. rRNA28S_1880R 5′- TTCGGTTCATCCCGCAGCGCCAGTTC-3′
5. rRNA28S_3647F 5′- AAACAAAGCATCGCGAAGG-3′
6. rRNA28S_3740R 5′- CGCTTCATTGAATTTCTTCACTT-3′
7. rRNA18S_930F 5′-GACGGCCGGGGGCATTCGTATTG-3′
8. rRNA18S_1050R 5′- CGACGGTATCTGATCGTCTTCGAACC-3′

### VIDISCA

VIDISCA was performed as described with some adaptations [6]. In short, cell debris and mitochondria were removed by centrifugation and residual DNA was degraded with 20 U TURBO™ DNase (Ambion). Nucleic acid isolation was performed as described by Boom *et al*.[23], elution in H_2_O with or without 10 µM rRNA-blocking oligonucleotides:

- 1-Morrna 5′ CTTTCGCTCTGGTCCGT 3′ –C6 [18S, nt. 977–1071]
- 2-Morrna 5′ CACTAATTAGATGACGAGG 3′–C6 [28S, nt. 3767–3785]
- 3-Morrna 5′ TGACATTCAGAGCACTGG 3′–C6 [28S, nt. 3679–3696]
- 4-Morrna 5′ GTTACTGAGGGAATCCTG 3′ –C6 [28S, nt. 72–89]
- 5-Morrna 5′ CACCAGTTCTAAGTCGG 3′–C6 [28S, nt. 3580–3596]

Reverse transcription was performed with 2.5 µg of random hexamers (Amersham Biosciences) or 2.5 µg non-ribosomal hexamers [8] and 200 U of Moloney murine leukemia virus reverse transcriptase enzyme (Invitrogen). After the RT reaction, second strand synthesis was performed with 5 U Klenow frament (3′ - 5′ exo-) (Westburg) and 7.5 U of RNase H (Amersham) followed by a phenol chloroform extraction and ethanol precipitation. The digestion was performed for 2h at 37°C by 10 U of *HinP1-I* (New England Biolabs) and 10U of *MseI* (New England Biolabs) restriction enzymes or only by 10U of *MseI* (New England Biolabs). Ligation of MSE and HINP anchors was performed as described [6]. In case of single *MseI* digestion a 2^nd^ MSE anchor was added (MID1-top-A 5′-GCCTCCCTCICGCCATCAGACGAGTGCGTA-3′; MID1-bottom-A 5′-TATACGCACTCGTCTGATGGCGCGAGGGAGGC-3′; Top-B 5′- GCCTTGCCAGCCCGCTCAGA-3′; Bottom-B 5′-TATCTGAGCGGGCTGGCAAGGC-3′). The first round of PCR amplification was performed with primers annealing to the anchors and covers 20 cycles, or 45 cycles in case only a single PCR was used. A second PCR was used to enhance the signal using primers that are extended at the 3′ with one nucleotide (either A, T, C, or G) so a total of 16 primer combinations. PCR fragments were visualized on 3% metaphor agarose gels (Cambrex), fragments of interest were cut from gel, purified with NucleoSpin® Extract II (Macherey-Nagel), cloned using TOPO TA cloning kit (Invitrogen) and sequenced with BidDye terminator reagents (Applied Biosystems). Data analysis was conducted with CodonCode Aligner software and BLAST.

### VIDISCA-454

**Figure 4:**
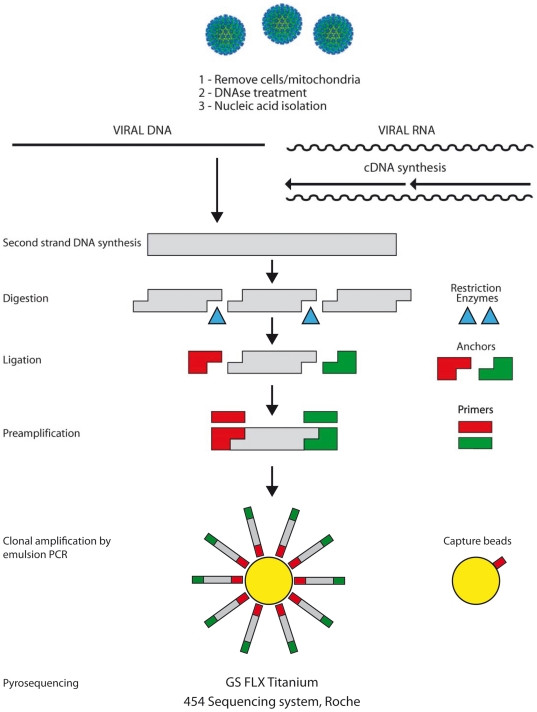
Schematic overview of VIDISCA-454.

VIDISCA was performed as described above with minor changes (Figure 4). Reverse transcription was performed with Superscript II (200 U, Invitrogen) in a mixture containing E.coli ligase (5 U, Invitrogen). The anchor ligation was performed with anchors, based on primer A with an identifier sequence (MIDs of 10 nt see GS FLX Shotgun DNA Library Preparation Method Manual) and 1 anchor containing primer B. In total 14 different identifier sequences were used, allowing 14 samples to be pooled. Amplification in a single PCR was performed with 0.4 µM of primer A-MID (5′- CGTATCGCCTCCCTCGCGCCATCAG -3′) and 0.4 µM of primer B (5′- CTATGCGCCTTGCCAGCCCGCTCAG -3′) with the following thermo-cycling profile: 1 cycle of 94°C for 5 min, 40 cycles of 94°C for 60 s, 55°C for 60 s and 72°C for 2 min, and 1 cycle of 72°C for 10 min. Of each sample 15 µl of product was loaded on a 1% agarose gel and 3 size regions were cut from gel: 200–300 bp, 300–500 bp and 500–700. Each size region was purified with NucleoSpin® Extract II (Macherey-Nagel). DNA was quantified with the Quant-iT**™** dsDNA Assay Kit on a Qubit fluorometer (Invitrogen). Emulsion PCR was performed according to the suppliers protocol (LIB-A SV emPCR kit, GS FLX Titanium PicoTiterPlate kit (70×75), GS FLX Titanium XLR 70 Sequencing kit (Roche)). Each emulsion PCR amplifies fragments of 14 different samples. Samples were run on a 4 regions Picotiterplate for the 454 Titanium system (per region 14 samples were run) and processed according to the emulsion small volume PCR protocol with 2 E6 beads per emulsion as input and 4 small volume emulsions per region (direct titration protocol). Sequence reads were assembled using the CodonCode software (www.codoncode.com) and the search for viral sequences was performed with the Blast tool of Genbank.
